# Changes in phenological events in response to a global warming scenario reveal greater adaptability of winter annual compared with summer annual arabidopsis ecotypes

**DOI:** 10.1093/aob/mcaa141

**Published:** 2020-07-29

**Authors:** Steven Footitt, Angela J Hambidge, William E Finch-Savage

**Affiliations:** 1 School of Life Sciences, Wellesbourne Campus, University of Warwick, Warwickshire, UK; 2 Department of Molecular Biology and Genetics, Boğaziçi University, Bebek, Istanbul, Turkey

**Keywords:** Arabidopsis, climate change, dormancy, flowering time, global warming, seedling emergence, summer annual, temperature adaptation, winter annual

## Abstract

**Background and Aims:**

The impact of global warming on life cycle timing is uncertain. We investigated changes in life cycle timing in a global warming scenario. We compared *Arabidopsis thaliana* ecotypes adapted to the warm/dry Cape Verdi Islands (Cvi), Macaronesia, and the cool/wet climate of the Burren (Bur), Ireland, Northern Europe. These are obligate winter and summer annuals, respectively.

**Methods:**

Using a global warming scenario predicting a 4 °C temperature rise from 2011 to approx. 2080, we produced F_1_ seeds at each end of a thermogradient tunnel. Each F_1_ cohort (cool and warm) then produced F_2_ seeds at both ends of the thermal gradient in winter and summer annual life cycles. F_2_ seeds from the winter life cycle were buried at three positions along the gradient to determine the impact of temperature on seedling emergence in a simulated winter life cycle.

**Key Results:**

In a winter life cycle, increasing temperatures advanced flowering time by 10.1 d °C^–1^ in the winter annual and 4.9 d °C^–1^ in the summer annual. Plant size and seed yield responded positively to global warming in both ecotypes. In a winter life cycle, the impact of increasing temperature on seedling emergence timing was positive in the winter annual, but negative in the summer annual. Global warming reduced summer annual plant size and seed yield in a summer life cycle.

**Conclusions:**

Seedling emergence timing observed in the north European summer annual ecotype may exacerbate the negative impact of predicted increased spring and summer temperatures on their establishment and reproductive performance. In contrast, seedling establishment of the Macaronesian winter annual may benefit from higher soil temperatures that will delay emergence until autumn, but which also facilitates earlier spring flowering and consequent avoidance of high summer temperatures. Such plasticity gives winter annual arabidopsis ecotypes a distinct advantage over summer annuals in expected global warming scenarios. This highlights the importance of variation in the timing of seedling establishment in understanding plant species responses to anthropogenic climate change.

## INTRODUCTION

Plants synchronize their life cycles with changes in the seasonal environment (phenology) (Donohue, 2014). The major phase changes in plant life cycles are flowering and germination, which mark the transitions to the reproductive and the vegetative phases, respectively. These phenological events are linked and both are known to be temperature driven (Springthorpe and Penfield, 2015; Burghardt *et al.*, 2016; Finch-Savage and Footitt, 2017; Penfield and MacGregor, 2017).

Meta-analysis of 20–50 years of multispecies flowering time data found it to have advanced by 1 d per decade in species responding to warming spring temperatures (Cook *et al.*, 2012). This makes understanding the impact of global warming on plant phenology crucially important as we address the resilience of both agricultural systems and native flora. A number of studies have highlighted the advancement of plant growth and flowering as spring becomes warmer (as reviewed in Parmesan and Hanley, 2015). Other studies have shown the impact of reduced winter chilling on major temperate fruit crop species such as *Malus*, *Pyrus* and *Prunus*, with higher winter and spring temperatures resulting in increased flower bud abscission, and poor flower quality, fruit set and yield (as reviewed in Atkinson *et al.*, 2013).

A model showing the impact of temperature on flowering time, seed set and dormancy in the *Arabidopsis thaliana* (L.) Heynh. ecotype Columbia (Col-0), which exhibits both winter and summer annual behaviour, was developed by Springthorpe and Penfield (2015). Increasing temperature accelerated flowering time to set seed development in an ambient temperature window coincident with a temperature-sensitive switch in dormancy. This resulted in seed shedding at a temperature range of 14–15 °C. Seeds produced below this range were more dormant and those above less dormant. This is a bet hedging strategy to produce seeds at a range of dormancy levels that will respond to conditions for optimum seedling emergence in the field (Springthorpe and Penfield, 2015). When seeds of obligate winter and summer annual *A. thaliana* ecotypes were matured along a thermal gradient in a global warming scenario, the critical temperature for switching from deep to shallow dormancy was in the range 14–16 °C (Huang *et al.*, 2018).

These two studies show that parental plasticity provides an indication of how plants adjust their phenology along a climate/temperature gradient but do not address their potential response over more than one generation to global warming. Auge *et al.* (2017) reasoned that although parental plasticity is a useful predictor of progeny behaviour in stable seasonal environments, this relationship might break down if conditions change in the next generation. At this point, the plasticity of the progeny to environmental signals is a better predictor of life cycle outcomes. The plasticity of the parent as evidenced by its ability to respond to within-generation environmental change has a direct bearing on the ability to adapt to trans-generational changes (for a review, see Auge *et al.*, 2017).

In a changing climate, populations with the greatest genetic variability will more readily adapt. This may take the form of the advancement in flowering time (Cook *et al.*, 2012; Springthorpe and Penfield, 2015), or through the spatial and temporal dispersal of seeds (De Casas *et al.*, 2015). Both aspects of seed dispersal influence the environments experienced by progeny. Spatial dispersal enables exploitation of more favourable environments by physically relocating seeds, while temporal dispersal in the form of seed dormancy cycling synchronizes the seedling emergence with environments favourable for successful seedling establishment. These strategies are reviewed by De Casas *et al.* (2015) and Finch-Savage and Footitt (2017).

Dormancy/germination is under strong environmental and genetic control. Secondary dormancy induction that occurs post-seed dispersal is by either low or high temperature depending on the climate to which populations are adapted (Footitt *et al*., 2011, 2018; Montesinos-Navarro *et al.*, 2012; Huang *et al.*, 2015). These responses to temperature are likely to alter life cycle timing as the climate warms, for example flowering time (Cook *et al.*, 2012), and in the life cycles of a wide range of animal species (Bradshaw and Holzapfel, 2008).

Work on *Alliaria petiolata* (M. Bieb.), whose seeds have simple dormancy requiring only exposure to low temperature for dormancy removal and seedling emergence, showed that global warming would result in reduced seedling emergence. However, as seeds emerging at higher temperatures have reduced dormancy and therefore a reduced requirement for low temperature, selection for lower dormancy would result in subsequent generations maintaining competitiveness (Footitt *et al.*, 2018). To understand temperature-driven changes in the relationship between flowering time and dormancy Marcer *et al.* (2018) surveyed 300 Iberian populations of *A. thaliana*. This revealed that as minimum temperature increases, early flowering and deeper seed dormancy were favoured, indicating that this life cycle phenotype would become more common.

Here, we address the impact of global warming under ‘natural’ seasonal conditions on the phenological plasticity of the two major phase transitions in the plant life cycle: flowering time and seedling emergence (Donohue, 2014). To do this, we adopted a unique approach involving reciprocal transplantation along a thermal gradient in a common garden. We established a thermal gradient of +4 °C in a thermogradient tunnel to produce a realistic global warming scenario for the experimental area between 2011–2013 and 2080 (Wurr *et al.*, 1996; Huang *et al.*, 2018). Using a combined global warming–common garden approach, we previously showed the potential for changes in seed maturation temperature to alter seed dormancy and germination behaviour in crop and weed members of the Brassicaceae (Footitt *et al.*, 2017; Awan *et al.*, 2018; Huang *et al.*, 2018).

Due to its wide geographical range and large number of ecotypes, Arabidopsis is an ideal indicator species for investigating the impact of global warming on plant phenology. This is relevant in the wider context of plant biology. Here, in a global warming scenario, we test the phenological plasticity of the respective obligate winter and summer annual arabidopsis ecotypes Cvi from the Cape Verdi islands in Macaronesia and Bur from the Burren in Ireland, Northern Europe that have adapted to hot/dry and cool/wet climates separated by 17 °C of latitude (Footitt *et al.*, 2013). Using seeds produced at opposite ends of a thermal gradient, we raised plants under reciprocal temperature conditions under winter and summer life cycles.

## MATERIALS AND METHODS

### Simulating a global warming scenario in a thermogradient tunnel

Experiments used *Arabidopsis thaliana* in a field-based thermogradient tunnel (Wurr *et al.*, 1996) to investigate the impact of temperature over two generations in a global warming scenario. Operation of this polyethylene tunnel (32 m long × 9 m wide) is described elsewhere (Wurr *et al.*, 1996; Footitt *et al.*, 2017; Huang *et al.*, 2018). The tunnel enabled plant growth under natural daylengths and with a high percentage (76 %) of natural levels of irradiance. The basic operation involves monitoring the temperature outside the tunnel, reacting to which an electronic climate control system operates fans generating opposing warmed and ambient air flows to maintain an air temperature gradient from ambient at one end of the tunnel to approximately ambient +4 °C at the other end (Wurr *et al.*, 1996). This represents a projected median emissions scenario for the local experimental area used in this work (West Midlands, UK) that indicates an increase in the summer mean temperature of 3.7 °C by 2080 (UK Climate Change Projections, 2014; http://ukclimateprojections.metoffice.gov.uk/). Continuous monitoring of air and soil temperatures along the tunnel enabled varying degrees of simulated climate warming depending on position along the tunnel. This established realistic seasonal and diurnal air and soil temperature fluctuations in the tunnel.

### Plant material and growth conditions

**Fig. 1.**
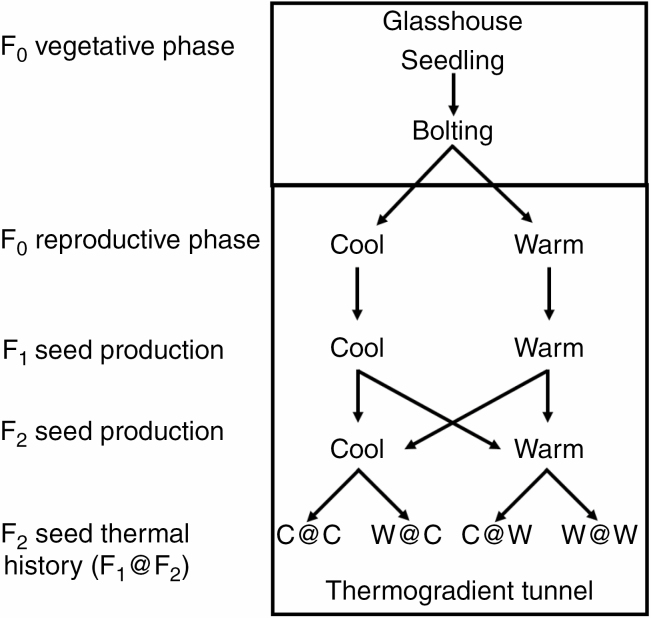
Seed thermal history. F_0_ seedlings were grown under glasshouse conditions then at bolting transferred to the thermogradient tunnel. F_0_ plants then produced F_1_ seeds at the cool (C) and warm (W) ends of the thermal gradient. F_1_ seedlings were grown under winter and summer annual life cycles at both ends of the thermal gradient, producing F_2_ seeds with contrasting thermal histories.

The experiments compared two arabidopsis ecotypes, Cape Verde Islands (Cvi; N8580) and Burren (Bur; N6643) that exhibit obligate winter and summer annual behaviour, respectively, at the experimental site used (Footitt *et al.*, 2013). In February 2011, non-dormant seeds of each ecotype were sown into compost (Levington F2/sand/vermiculite at a ratio of 6:1:1) in P24 cellular trays (24 cells, each 5 × 5 × 5 cm) held in capillary matting-lined seed trays. Seedlings were grown in a temperature-controlled glasshouse (23/17 °C, 16/8 h, light/dark) to bolting and then transferred to the thermal gradient as described in Huang *et al.*, (2018) (Fig. 1). Plants were placed at the cool/ambient end and the warm end of the tunnel (denoted as positions C and W) representing current temperature and the presumptive temperature for 2080. Harvesting of mature F_1_ seeds was from the 21 to 26 April 2011 by hand threshing, followed by equilibration at 15 % relative humidity/15 °C for 7 d to produce an equilibrium moisture content of 5–7 % on a dry weight basis. Seeds were stored at −80 °C in sealed tubes. This strategy enabled completion of the reproductive phase (bolting to mature seed) under the conditions of a winter annual life cycle.

In 2012, F_1_ seeds produced above at the C and W end of the thermal gradient were surface sterilized in a 0.125 % sodium hypochlorite solution (household bleach: 5 % sodium hypochlorite, diluted to 2.5 % v/v) for 5 min and then washed three times with distilled water. Seeds (≥200 from each cohort) were then plated onto a sterile nylon mesh (mesh size 125 μm; Clarcor UK, UK) held in Petri plates containing 0.7 % agarose and half-strength Murashige and Skoog (1/2 MS) salts at pH 5.8. Dormancy was broken by nicking the seed coats with a syringe needle. After sealing with micropore tape, then sealing in freezer bags and wrapping in aluminium foil, the plates were incubated at 5 °C/dark for 3 d then transferred to constant light at 15 °C. Following germination, seedlings were transplanted at the first true leaf stage to compost in trays as above in the thermogradient tunnel. This gave 24 seedling from each C and W cohort at each end of the thermal gradient.

Seedlings of both ecotypes grown in the thermogradient tunnel experienced winter and summer annual life cycles (hereafter denoted as WLC and SLC, respectively), as described below, under ambient temperature at the cool end and those predicted for 2080 at the warm end of the tunnel. Seedlings (*n* = 24) from F_1_ seeds produced at the cool end were grown to maturity at both positions C and W (denoted respectively as C@C and C@W) with one tray (*n* = 24) at each position. Similarly, at the same time, seedlings of F_1_ seeds produced at the warm end were grown to maturity at both positions. This gives the intergeneration temperature combinations of C@C, W@C, C@W and W@W (Fig. 1). In the WLC, seedling were transplanted to the tunnel on 19 November 2012, and in the SLC on 17 May 2013. At bolting, each plant was isolated using an Aracon (Arasystem, Belgium) to prevent cross-pollination and to facilitate seed collection. When two-thirds of the siliques on the plants in a tray had turned yellow, watering stopped to allow plants to dry for 7 d. At harvest, each plant was dried in a paper bag for 7 d at 15 °C/15 % relative humidity. After threshing and cleaning, F_2_ seeds were sealed in tubes, and stored at –80 °C. A number of parameters were recorded as components of fitness for each plant during the life cycle and post-harvest. These included the phenological measures days from transplanting to bolting (inflorescence extended to 1 cm) and number of rosette leaves at bolting, and plant height at harvest as a measure of size. Further direct measures of fitness were plant dry weight including flower and silique tissue following seed collection, and seed yield. Plant dry weight was determined after drying at 80 °C for 48 h.

### Germination analysis of F_2_ seeds

Seeds were surface sterilized as above then plated in three replicates of 40 seeds into 12 × 8 cm boxes (Stewart Plastics Ltd, UK) containing two pieces of 3MM chromatography paper and 8 mL of liquid. For a single ecotype, this enabled direct comparison of one replicate for each of the four temperature combinations in a single box.

Germination of fresh seeds of both ecotypes was tested in the presence of 50 μm gibberellin_4 + 7_ (GA_4 + 7_ was dissolved in 100 µL of 0.1 m KOH before preparing stock solution) in 1.7 mm citric acid/3.3 mm K_2_HPO_4_ buffer (pH 5.0) or a buffer control in the light at 20 °C. Germination was recorded as emergence of the radicle through the testa and micropylar endosperm over 28 d.

### Seedling emergence of F_2_ seeds in a global warming scenario in a thermogradient tunnel

We investigated the response of seedling emergence in a realistic global warming scenario along the thermal gradient. Burial of seeds of both ecotypes produced under all temperature regimes during the WLC was in pots to represent shedding to soil in late spring consistent with this annual life cycle in order to investigate seed behaviour in the seed bank. Actual burial dates were, Bur on 10 June 2013 (C@W and W@W) and 21 June 2013 (C@C and W@C) and for Cvi on 23 May 2013 (C@W and W@W) and 21 June 2013 (C@C and W@C). The delayed dates resulted from delayed flowering and seed maturation at the cool end of the thermal gradient. Soil was disturbed in each pot every 2 weeks to expose seeds to light. Seedling emergence was recorded and seedlings removed weekly, more often during peak emergence periods, until October 2014.

Burial was at three positions in the thermogradient tunnel designated as cool, medium and warm. The cool and warm positions were those used for plant growth, with the medium position equidistant between the two. The soil temperature gradient between the cool and warm ends of the tunnel was 2.5 °C. Three biologically independent replicates of 500 seed were used. Full experimental details for the seedling emergence trial are in Footitt *et al.* (2020).

### Data analysis

Vegetative and reproductive growth data were analysed using one-way analysis of variance (ANOVA) to test the impact of intergenerational temperature regimes on each variable. Two-way ANOVA was used to test for interaction of temperature regimes on biomass. In one-way ANOVA, Bonferroni correction was used and multiple comparisons for significance used Tukey’s range test. Flowering time, temperature from transplanting to bolting, temperature over 30 d prior to harvest, rosette leaf number at bolting, and plant height, dry weight and seed yield at harvest were analysed by one-way ANOVA separately for each life cycle and ecotype to determine the impact of the intergenerational temperature regimes (e.g. C@C). Final germination and seedling emergence is shown as the mean with the 95 % confidence interval. In the seedling emergence trial, seeds were buried at different times (see above) due to the impact of the thermal gradient on flowering time and seed development. To account for this, analysis of seedling emergence took the final burial date for each ecotype as the starting point for the analysis. Analysis was performed by converting total emergence to December 2013 to 100 % and calculating time to 50 % emergence of the total emerged seedlings by performing Probit transformation of the data and linear regression analysis of each replicate. Two-way ANOVA was performed to detect interaction between the global warming scenario (soil temperature) and the intergenerational temperature regimes separately on each ecotype. This was followed by one-way ANOVA to detect separately significant effects of the global warming scenario (soil temperature) and the intergenerational temperature regimes on each ecotype. Statistical analysis used Excel and the Real Statistics Resource Pack software (Release 6.8) (Zaiontz 2020). Statistical outputs of these analyses are given in Supplementary data, Data S1.

## RESULTS

Overall, there were large differences between the timing of bolting between ecotypes in a WLC, but timings were very similar in an SLC. Tunnel position and life cycle timing also had subsequent significant impacts on plant growth. There were also significant effects of ecotype and tunnel position on seedling emergence in a WLC. Detailed effects are given below for each ecotype. In figures and tables, the designations such as C@W and variations thereof refer to the seed thermal history. The first letter refers to the tunnel position (temperature) during F_1_ seed production (here Cool). The second letter refers to tunnel position during F_2_ seed production (here Warm) (refer to Fig. 1). It should also be noted that two temperature designations are used in descriptions of F_2_ seed production. These are the mean temperature from transplanting to bolting and mean seed maturation temperature (refer to Supplementary data Tables S1 and S2).

### F_1_ flowering time

Flowering marks the transition from the vegetative to the reproductive state, and is regulated by pathways that sense changes in temperature and photoperiod (Springthorpe and Penfield, 2015). Here we monitored flowering time and rosette leaf number at that time to evaluate the impact of the global warming scenario on the phenology of obligate winter and summer annual arabidopsis ecotypes during WLC and SLC.

#### Bur ecotype.

**Fig. 2.**
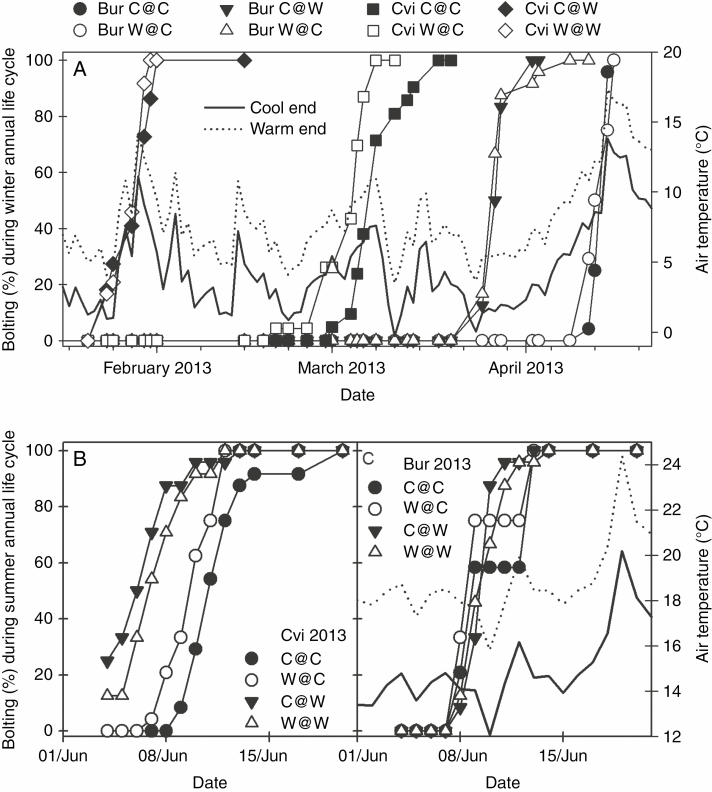
Bolting times of Bur and Cvi ecotypes grown in winter and summer annual life cycles in a global warming scenario. Plants of each intergenerational temperature regime were grown at the cool and warm ends of the thermal gradient. For each plant, bolting time was recorded when the floral bolt was 1 cm in height. (A) Bolting times during a winter annual life cycle. Bolting times during a summer annual life cycle were similar in both ecotypes as shown in (B) Cvi and (C) Bur. Air temperature at the cool and warm ends of the thermal gradient was recorded at a height of 50 cm.

**Fig. 3.**
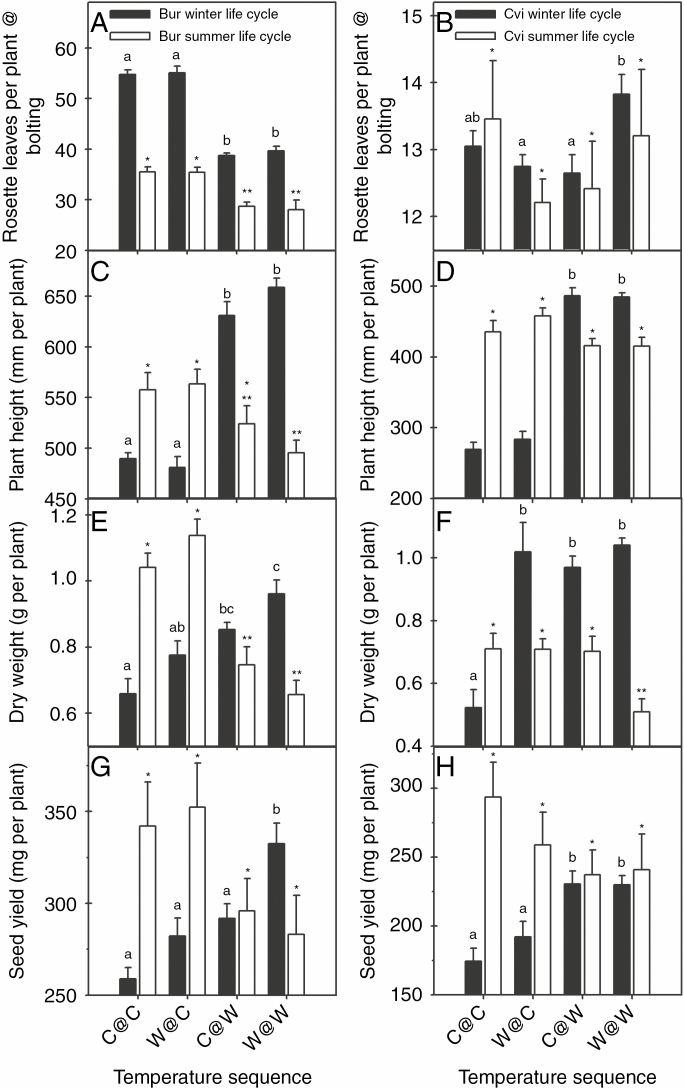
Phenotypic differences between plants produced in each intergeneration temperature regime in winter and summer life cycles in a global warming scenario. For all plants, the following characters were measured: the number of rosette leaves at bolting (A and B) and, at harvest, individual plant height (C and D), dry weight (E and F) and seed yield (G and H). In the winter and summer life cycles in each intergenerational temperature regime, all data are the means ± s.e. of 24 plants (*n* = 24). Analysis was by one-way ANOVA with Bonferroni correction followed by multiple comparisons for significance using Tukey’s range test. The ANOVA-generated *F* statistics are as follows; rosette leaf number winter life cycle (WLC) Bur (*F*_3, 92_ = 87, *P* < 0.0001) and Cvi (*F*_3, 84_ = 4.81, *P* = 0.0038); and summer life cycle (SLC) Bur (*F*_3, 92_ = 10.28, *P* < 0.0001) and Cvi (*F*_3, 92_ = 0.6, *P* = 0.56). Plant height, WLC, Bur (*F*_3, 92_ = 81, *P* < 0.0001) and Cvi (*F*_3, 90_ = 148, *P* < 0.0001); SLC Bur (*F*_3, 92_ = 4.09, *P* < 0.0088) and Cvi (*F*_3, 92_ = 2.49, *P* = 0.064). Plant dry weight, WLC, Bur (*F*_3, 92_ = 10.52, *P* < 0.0001) and Cvi (*F*_3, 90_ = 16.36, *P* < 0.0001); SLC, Bur (*F*_3, 92_ = 23.19, *P* < 0.0001) and Cvi (*F*_3, 92_ = 5.09, *P* < 0.0026). Seed yield, WLC Bur (*F*_3, 92_ = 11.75, *P* < 0.0001) and Cvi (*F*_3, 90_ = 8.58, *P* < 0.0001); and SLC Bur (*F*_3, 92_ = 2.418, *P* = 0.071) and Cvi (*F*_3, 92_ = 1.213, *P* = 0.309). Temperature regimes for ecotype × life cycle combinations in a WLC identified by a different letter are significantly different. In an SLC, those identified by * or ** are significantly different.

When the summer annual ecotype Bur experienced a WLC, bolting occurred when air temperature was consistently ≥5 °C. Floral meristems first bolted on 25 March 2013 at the warm end of the thermal gradient (tunnel position W) followed 16 d later (11 April) at the cool end of the gradient (tunnel position C) (Fig. 2A). The difference in days from transplanting to bolting was significant (*F*_3, 92_ = 428, *P* < 0.0001) between the cool and warm ends of the gradient (with bolting time 11 % less at the warm end) (Table 1). Mean temperature was also significantly different over this period between the cool and warm end of the tunnel (*F*_3, 92_ = 34 565, *P* < 0.0001) (Supplementary data Table S1). The delay in flowering at the cool end of the gradient led to a greater rosette leaf number (28 %) compared with that at the warm end (Fig. 3A). (*F*_3, 92_ = 87, *P* < 0.0001). When experiencing its ‘natural’ SLC, bolting started on 11 June 2013 and on 14 June 2013 at the warm and cool ends, respectively (Fig. 2C), with the mean bolting time significantly earlier by 2 d at the warm end (*F*_3, 92_ = 35, *P* < 0.0001). Mean temperature was also significantly different over this period (*F*_3, 92_ = 27 565, *P* < 0.0001), with rosette leaf number 20 % less at the warm end (*F*_3, 92_ = 10, *P* < 0.0001) (Fig. 3A; Table 1) of the thermal gradient. In each life cycle, there was no significant impact of the temperature experienced during F_1_ seed development. The difference in mean temperature to bolting between the cool and warm ends of the thermal gradient for Bur during the WLC and SLC was 3.25 and 4.06 °C, respectively, giving an advancement in flowering time in response to global warming of 4.92 and 0.49 d °C^–1^, based on the mean bolting time (Table 1; Supplementary data Table S1).

**Table 1.** Days to bolting of the floral meristem in the obligate winter and summer annual arabidopsis ecotypes Cvi and Bur

Temperature regime	Days to bolting of the floral meristem			
	Winter life cycle		Summer life cycle	
	Bur (*P* < 0.001)	Cvi (*P* < 0.05)	Bur (*P* < 0.001)	Cvi (*P* < 0.05)
**C@C**	145.5 ± 0.2^a^	109.4 ± 1.0^a^	26.2 ± 0.2^a^	26.0 ± 0.6^a^
**W@C**	145.0 ± 0.3^a^	104.7 ± 0.8^b^	25.9 ± 0.2^a^	24.1 ± 0.3^b^
**C@W**	129.1 ± 0.5^b^	70.6 ± 0.6^c^	23.8 ± 0.2^b^	20.6 ± 0.5^c^
**W@W**	128.9 ± 0.7^b^	70.4 ± 0.5^c^	24.0 ± 0.3^b^	21.5 ± 0.5^c^

Time from transplanting to bolting when experiencing winter and summer annual life cycles in a thermal gradient tunnel. The temperature regime represents the temperature for F_1_ and F_2_ seed production (F_1_@F_2_, C = cool and w = warm; refer to Fig. 1). Data represent the mean ± s.e. In single columns only, data followed by the same letter are not significantly different as follows: winter life cycle Bur (*F*_3, 92_ = 428, *P* < 0.0001), Cvi (*F*_3, 86_ = 852, *P* < 0.0001); summer life cycle Bur (*F*_3, 92_ = 35, *P* < 0.0001), Cvi (*F*_3, 92_ = 28, *P* < 0.0001). Analysis was by one-way ANOVA with Bonferroni correction followed by multiple comparisons for significance using Tukey’s range test. Information relates to data in Fig. 1.

#### Cvi ecotype.

When the winter annual ecotype Cvi experienced its ‘natural’ winter annual life cycle, the difference in days to bolting was significant (*F*_3, 86_ = 852, *P* < 0.0001) between the cool and warm ends of the thermal gradient. Bolting started on 24 January 2013 at the warm end and on 20 February 2013 at the cool end (Fig. 2A). At the cool end, there was a significant effect of temperature experienced during seed development, with F_1_ plants from seeds produced at the warm end bolting 5 d earlier than those produced at the cool end (*F*_3, 86_ = 852, *P* < 0.0001) (Table 1). In an SLC, bolting time between Cvi plants at opposite ends of the thermal gradient was also significantly different (*F*_3, 92_ = 28, *P* < 0.0001) (Fig. 2B; Table 1). Again plants from F_1_ seeds produced at the warm rather than the cool end of the gradient bolted significantly earlier (2 d) at the cool end (*F*_3, 92_ = 28, *P* < 0.0001). In the WLC, rosette leaf number at bolting was significantly higher when F_1_ and F_2_ seed production was at the same position on the thermal gradient (Fig. 3B) (*F*_3, 84_ = 4.8, *P* = 0.0038), but this was unlikely to be physiologically significant. In the SLC, there was no significant difference in rosette leaf number (*F*_3, 92_ = 0.68, *P* < 0.56). The difference in mean temperature to bolting along the thermal gradient in Cvi during the WLC and SLC was 3.6 and 4.0 °C, respectively, giving an advancement in flowering time of 10.1 and 1.0 d °C^–1^ (Table 1; Supplementary data Table S1).

### Impact of temperature on plant height, biomass and seed yield

**Fig. 4.**
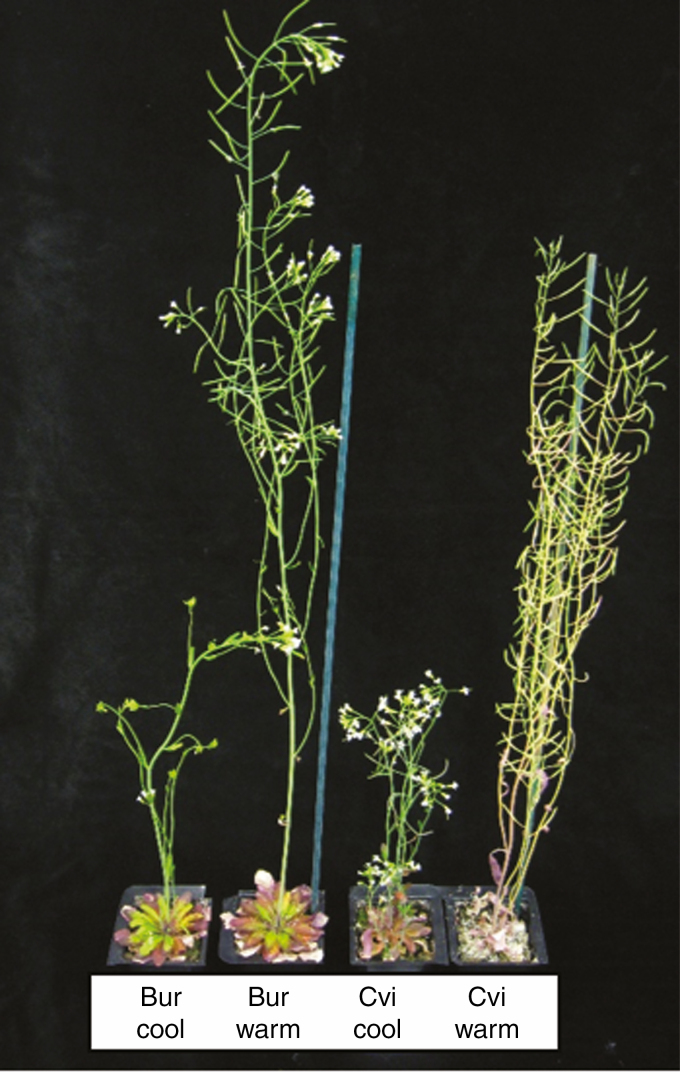
Differences in overall plant growth during a winter life cycle resulting from conditions of the global warming scenario. Seedling of the Bur and Cvi ecotypes were transplanted into position on 19 November 2012 and placed at opposite ends of the thermal gradient. The thermal history of the plants is C@C and W@W. The image was recorded on 25 April 2013.

At harvest, plant height (a measure of size), biomass (dry weight including the valves and floral tissue collected post-seed threshing) and seed yield (measures of fitness) were determined to evaluate the impact of the global warming scenario on arabidopsis during WLC and SLC. In the WLC, the impact of the global warming scenario on flowering time resulted in large differences in overall progression of plant growth (Fig. 4).

#### Bur ecotype

*Height.* In Bur, only the F_2_ temperature had a significant effect on plant height which was >24 % (*F*_3, 92_ = 81, *P* < 0.0001) greater at the warm than the cold end during the WLC. In the SLC, height was 9 % (*F*_3, 92_ = 4.09, *P* = 0.0088) less at the warm end (Fig. 3C).

*Biomass.* Temperature regime had a significant impact on aerial plant biomass (Fig. 3E, F). During the WLC, plants had a significantly 20 % greater biomass when F_2_ seed production was at the warm end, and was >11 % in W@W (both F_1_ and F_2_ at the warm end) (*F*_3, 92_ = 10, *P* < 0.0001). This reflected a progressively greater accumulation in biomass (Fig. 3E). In the SLC, Bur biomass was significantly less (35 %) when F_2_ seed production was at the warm end (*F*_3, 92_ = 230, *P* < 0.0001).

*Seed yield.* During the WLC, there was a significant intergenerational impact of temperature on seed yield (reproductive output) which was 12 % greater in W@W compared with C@W (*F*_3, 92_ = 11, *P* < 0.0001) (Fig. 3G). In the SLC, seed yield was 16 % less at the warm end of the gradient, but this difference was not statistically significant (*F*_3, 92_ = 2.4, *P* = 0.071). The relationship between biomass and seed yield is linear in both life cycles (*R* = 0.9504).

**Fig. 5.**
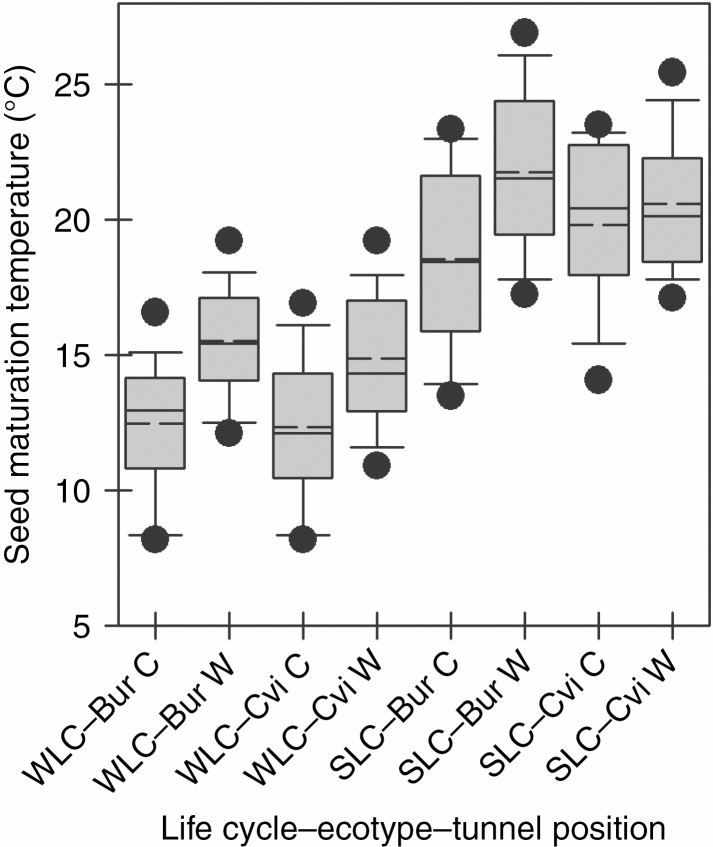
Seed maturation temperatures of F_2_ seeds of Bur and Cvi ecotypes during winter and summer life cycles in a global warming scenario. Box plots show the mean (dashed line) and median (solid line) temperature. The box shows the 25th and 75th percentiles, the whiskers the fifth and 95th percentile, and the filled circle the outliers. Mean temperatures were determined over the maturation period, which was 30 d prior to harvest in all but Cvi under the C@W and W@W regimes, which were 26 and 27 d long, respectively. Winter life cycle and summer life cycle are abbreviated as WLC and SLC.

*F*_*2*_ *seed maturation temperature.* The mean temperature experienced during seed maturation was significantly higher for plants grown at the warm end of the thermal gradient in both WLC (*F*_3, 116_ = 18, *P* < 0.0001) and SLC (*F*_3, 116_ = 11, *P* < 0.0001) (Fig. 5; Supplementary data Table S2). The temperature difference between the two ends of the thermal gradient was 3.0 °C in the WLC and SLC.

**Fig. 6.**
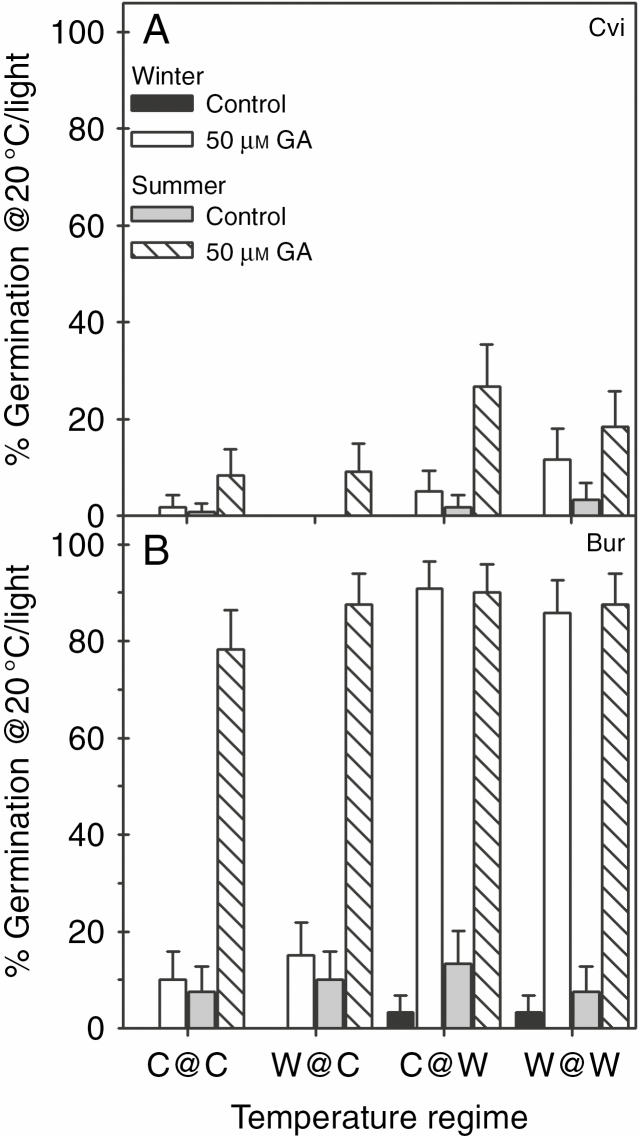
Germination of F_2_ seeds produced in winter and summer life cycles in a global warming scenario. Seeds of (A) Cvi and (B) Bur produced in each intergenerational temperature regime were incubated at 20 °C in the light in the presence of 50 μm gibberellin_4 + 7_ at pH 5.0 or in a buffer control at pH 5.0. Germination was recorded as emergence of the radicle through the testa and micropylar endosperm over 28 d. Missing columns indicate where seeds were too dormant to germinate. Data are the mean ± 95 % confidence interval, *n* = 3.

*F*_*2*_ *seed dormancy.* Bur seeds produced at the warm end in the WLC responded significantly to GA, while those produced at the cool end were highly dormant (*F*_3, 8_ = 235, *P* < 0.0001) (Fig. 6B). In the SLC, there was a high response to GA in all temperature regimes which did not differ significantly (*F*_3, 8_ = 0.55, *P* = 0.657). Viability of seeds from WLC and SLC produced under all temperature regimes was >97 %.

#### Cvi ecotype

*Height.* In the WLC, only the F_2_ temperature had a significant effect on plant height which was 70 % greater at the warm end than at the cool end (*F*_3, 90_ = 4.09, *P* < 0.0001). In the SLC, there was no significant difference (*F*_3, 92_ = 2.49, *P* = 0.065) (Fig. 3D).

*Biomass.* A significant intergenerational effect was detected when interaction between F_1_ and F_2_ temperatures was compared (two-way ANOVA *F*_1, 90_ = 21, *P* < 0.0001). This was only seen in the WLC at the cool end of the gradient, with biomass 53 % greater when F_1_ seeds were produced under warm conditions (*F*_3, 90_ = 16, *P* < 0.0001) (Fig. 3F). This increase was consistent when F_2_ seed production was under warm conditions. In the SLC, biomass was significantly less (27 %) when both generations (W@W) were produced at the warm end compared with the cool end, representing an intergenerational effect (*F*_3, 92_ = 5.09, *P* = 0.0026).

*Seed yield.* In the WLC, growth at the warm end resulted in a significantly greater (23 %) seed yield in the F_2_ seed (*F*_3, 90_ = 8, *P* < 0.0001) (Fig. 3H). There was no impact of temperature on seed yield in the SLC (*F*_3, 92_ = 1.2, *P* = 0.309) although at the warm end of the gradient seed yield was 13 % smaller. Seed yield and biomass are not correlated in WLC (*R* = 0.74) or SLC (*R* = 0.46).

*F*_*2*_ *seed maturation temperature.* The mean temperature was significantly higher for plants grown at the warm end of the thermal gradient in the WLC (*F*_3, 116_ = 10, *P* < 0.0001) but not significant in the SLC (*F*_3, 111_ = 0.85, *P* = 0.46) (Fig. 5; Supplementary data Table S2). The temperature difference between the cool and warm ends of the gradient was 2.5 °C in the WLC and 0.86 °C in the SLC.

*F*_*2*_ *seed dormancy.* Dormancy in Cvi seeds produced in both WLC and SLC was so deep that dormancy comparisons (ability to germinate) with Bur had to be evaluated based on their sensitivity to GA. In Cvi, the response to GA was greater in seeds from the SLC, with those from the C@W regime responding significantly more than those of the C@C regime (*F*_3, 8_ = 4.89, *P* = 0.032) (Fig. 6A). In the WLC, the response to GA is significant (*F*_3, 8_ = 5.28, *P* = 0.026). Seed viability was >98 % (WLC) and >97 % (SLC).

### F_2_ seedling emergence

**Fig. 7.**
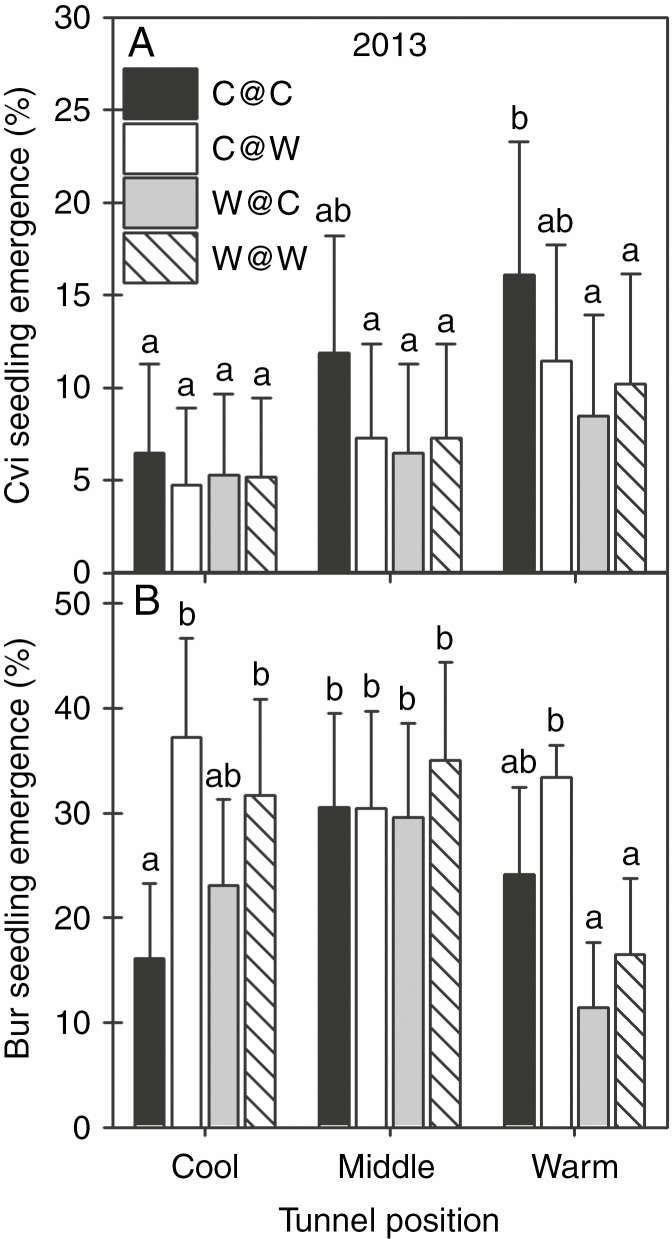
Seedling emergence of buried F_2_ seeds of Cvi and Bur produced in a winter life cycle in a global warming scenario. Final percentage seedling emergence in 2013 of (A) Cvi and (B) Bur seeds produced under different intergenerational temperature regimes. Analysis was by one-way ANOVA with Bonferroni correction followed by multiple comparisons for significance using Tukey’s range test. The level of significance for Cvi was *F*_11, 24_ = 5.48, *P* < 0.00025 and for Bur was *F*_11, 24_ = 6.03, *P* = 0.00012. For each ecotype, analysis covers all intergenerational and soil temperature combinations. Combinations identified by a different letter are significantly different. For each intergenerational temperature, *n* = 3 at each position on the thermal gradient.

F_2_ seeds of Bur and Cvi produced in the WLC were buried to simulate spring seed dispersal at three positions (cool/ambient, middle and warm) along the thermal gradient. Seedling emergence in both ecotypes occurred in late summer to early autumn in both years. Overall, seedling emergence was greater in Bur (Fig. 7). Seedling emergence in both ecotypes was greater in 2013 than in 2014 (see Supplementary data Fig. S1). Along the thermal gradient, Cvi emergence increased with soil temperature while in Bur emergence was less at the warm end (Fig. 7A, B). In Cvi, seeds from the C@C regime had the highest emergence of all the intergenerational temperature regimes at each tunnel position, and this was significant at the warm end (*F*_11, 24_ = 5.48, *P* < 0.00025). In Bur, there were significant differences (*F*_11, 24_ = 6.03, *P* = 0.00012) between the generally high emergence in the middle of the thermal gradient compared with that at the extremes. F_2_ seeds produced at the warm end of the gradient had significantly lower emergence (approx. 50 % lower) compared with emergence at other positions (Fig. 7B).

### F_2_ seedling emergence timing (SET)

**Fig. 8.**
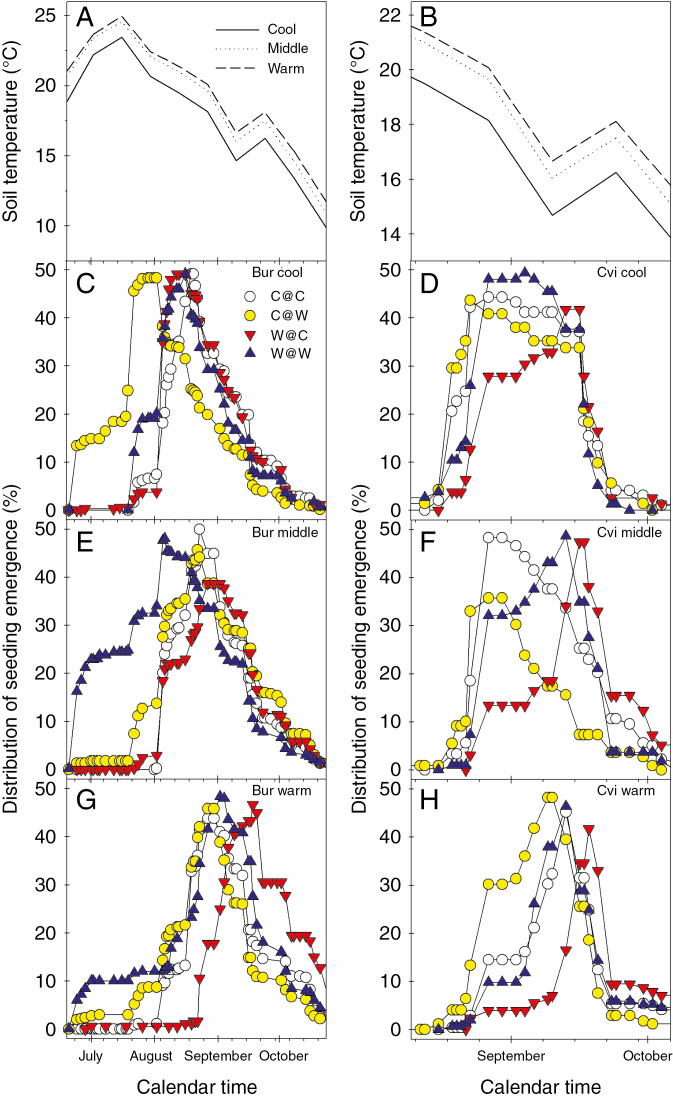
Seedling emergence timing (SET) of F_2_ seeds, produced in a winter life cycle, in a global warming scenario. Seed burial was in May and June 2013 (see the Materials and Methods for details) to mimic seed dispersal at the end of a winter life cycle. Soil temperature at seed depth along the thermal gradient is shown during the Bur (A) and Cvi (B) seedling emergence periods. The distribution of emergence over time is the positive accumulation to 50 % emergence followed by the negative accumulation to show the population response. Data represent the mean of three replicates. The SET response of Bur and Cvi seeds from each intergenerational temperature regime is shown for the cool (C and D), middle (E and F) and warm (G and H) positions along the thermal gradient.

The intergenerational temperature regimes had an impact on SET. This was visualized by setting total emergence for each intergenerational temperature regime and tunnel position in December 2013 to 100 %; then plotting the distribution of emergence over time as the positive accumulation to 50 % emergence followed by the negative accumulation to show the population response (Fig. 8). In Bur, SET started in July when soil temperature was increasing and continued through to the end of October as soil temperature declined (Fig. 8A, C, E and G). In contrast, in Cvi, the onset of SET was in August when soil temperature was declining, and persisted until October (Fig. 8B, D, F and H). The two ecotypes have significantly different SET peaks across the thermal gradient (*F*_1, 70_ = 26.98, *P* < 0.0001) (Supplementary data Table S3). However, at the warm end, Bur SET is significantly different from Bur SET at the cool end, but not significantly different from Cvi SET all along the gradient (*F*_5, 66_ = 14.11, *P* < 0.0001) (Supplementary data Table S3).

#### Bur SET.

In Bur, first emergence was from F_2_ seeds produced under warm conditions (Fig. 8C, E and G). At each position along the tunnel, the time from first to last peak in SET was 21–27 d (Table 2). Ranking peak SET from first to last for Bur (C@W < W@W < W@C < C@C at the cool end; W@W < C@C < C@W < W@C in the middle; and C@W <C@C = W@W<W@C at the warm end) indicated no strong pattern to the order of emergence (Table 2). However, at the warm end, the order of SET was the same as seen in Cvi (see below). Overall, increasing soil temperature delayed peak SET for each intergenerational temperature regime (Table 2). The time difference between the peak in SET along the thermal gradient was shortest when F_1_ and F_2_ seed production was at the same position in the tunnel (C@C and W@W) (Table 2).

**Table 2.** Date of 50 % seedling emergence along the thermal gradient

Inter generational temperature regime	Tunnel position			Difference in SET (d) between tunnel positions		
	Cool	Middle	Warm	C>M	M>W	C>W
Bur winter life cycle: spring burial						
C@C	22/8/2013	24/8/2013	30/8/2013	2	6	8
C@W	30/7/2013	26/8/2013	27/8/2013	27	1	28
W@C	17/8/2013	1/9/2013	17/9/2013	15	16	31
W@W	16/8/2013	5/8/2013	30/8/2013	–11	25	14
Difference in SET (d) at each tunnel position	23	27	21			
Cvi winter life cycle: spring burial						
C@C	29/8/2013	2/9/2013	13/9/2013	4	11	15
C@W	26/8/2013	28/8/2013	7/9/2013	2	10	12
W@C	10/9/2013	15/9/2013	19/9/2013	5	4	9
W@W	04/9/2013	7/9/2013	11/9/2013	3	4	7
Difference in SET (d) at each tunnel position	15	18	12			

Seedling emergence of Bur and Cvi seeds produced under different intergenerational temperature regimes. Burial of seeds produced in a winter annual life cycle in the second generation was at three positions in a thermal gradient tunnel. Dates indicate the mean point for 50 % seedling emergence. The time of 50 % emergence was determined by performing Probit transformation of the data and linear regression analysis of each replicate (*n* = 3). In the intergeneration temperature regime, C = cool and w = warm. Difference in SET for tunnel position is the period (d) between the first and last peak. Difference in SET for intergenerational temperature regime is the days between the respective peaks at the cool (C), middle (M) and warm (W) positions

Increasing soil temperature (tunnel position) significantly delayed peak SET in Bur by 20 d (*F*_2, 33_ = 8.55, *P* = 0.001) across all intergenerational temperature regimes (Supplementary data Table S4). In individual regimes, seeds produced under C@W (*F*_2, 6_ = 8.65, *P* < 0.016) and W@C (*F*_2, 6_ = 5.21, *P* < 0.048) had peak SET significantly delayed at the warm end compared with the cool end (Supplementary data Table S5).

#### Cvi SET.

Ranking peak SET from first to last revealed that seeds from plants experiencing different intergenerational temperature regimes always had the earliest and latest peak in SET (C@W < C@C < W@W < W@C at the cool and middle positions, and C@W < W@W < C@C < W@C at the warm end) (Table 2). When F_1_ seed production was under warm conditions, SET peaks were later by up to 15 d at the cool end of the thermal gradient (Fig. 8B; Table 2). Seeds produced at the same position on the thermal gradient over two generations had peak SET intermediate to those experiencing temperature switching. The shortest periods between peak SET along the thermal gradient were when F_1_ seed production was under warm conditions (Table 2). The time from the first to last peak in SET decreased to 12 d at the warm end of the tunnel in comparison with the cool and middle positions (Table 2).

Both soil temperature and growth regime had significant impacts on peak SET. Peak SET was significantly delayed by 10 d at the warm end compared with the cool end (*F*_2, 33_ = 6.97, *P* = 0.0029). Across all soil temperatures, seeds from the C@W regime had a peak SET significantly earlier by 15 d than those from W@C (*F*_3, 32_ = 8.2, *P* = 0.0003) (Supplementary data Table S4).

## DISCUSSION

In this study we investigated the impact of global warming on the life cycles of obligate winter (Cvi) and summer (Bur) annual ecotypes of arabidopsis ecotypes during winter and summer annual life cycles using a realistic global warming scenario in a common garden. F_1_ seeds were produced under a global warming scenario representing the ambient thermal environment (2011) and that predicted for approx. 2080 (ambient +3.7 °C) (Huang *et al.*, 2018). Each F_1_ cohort then experienced WLC and SLC to produce the F_2_ generation in our global warming scenario. In response to global warming, phenological plasticity was examined and this revealed intergenerational effects of temperature on both major phase transitions in the plants life cycle, i.e. flowering and seedling emergence.

### Flowering time

In the WLC, Cvi flowered 6–8 weeks earlier than Bur at both ends of the thermal gradient. In the SLC, this difference was only 3 d at the warm end, with no difference at the cool end. This difference in flowering time in the WLC results from the vernalization-independent late flowering phenotype of Bur driven by late flowering loci other than *FLOWERING LOCUS C* (*FLC*), because in Bur *FLC* is a null allele (Werner *et al.*, 2005). This mutation may drive the summer annual behaviour of Bur observed by Ratcliffe (1976) in its native habitat.

Significant differences in the time to bolting in both ecotypes in the WLC and SLC revealed differences in phenological plasticity of flowering time in response to global warming. In Bur, flowering time advanced by 4.92 and 0.49 d °C^–1^ in the respective WLC and SLC, while in Cvi it advanced by 10.1 and 1.0 d °C^–1^. This greater plasticity of the obligate winter annual ecotype Cvi shows that it is more adaptable to future global warming than the summer annual Bur which may reflect their adaptation to warm/dry (Cvi) and cool/wet (Bur) climates (Footitt *et al.*, 2013; Finch-Savage and Footitt, 2017). Differences in intergenerational temperature had no impact on flowering time in Bur. However, in Cvi, warm conditions during F_1_ seed development significantly advanced flowering time during F_2_ seed production at the cool end of the thermal gradient in both the WLC and SLC, indicating the operation of a thermal memory. The advance of flowering time in Cvi in the WLC (W@C) resulted in significantly greater biomass and a larger but non-significantly greater seed yield. In Bur, seed yield was less under warm summer conditions, as seen previously (Huang *et al.*, 2014). This indicates increased fitness and bet hedging potential in Cvi compared with Bur. In the Col-0 ecotype, ancestral heat stress also increased fitness and accelerated flowering (Whittle *et al.*, 2009; Migicovsky *et al.*, 2014). The mechanism underlying this thermo-memory that promotes reproduction and fitness by accelerating flowering was identified by Liu *et al.* (2019). This mechanism may contribute to other intergenerational temperature effects seen here, such as greater seed yield (Bur) and changes in biomass (Cvi).

The relationship between biomass and seed yield also differs between the two ecotypes. In Bur, these measures of fitness are positively correlated, but not in Cvi. The *FLC* null allele of Bur delays flowering time in the WLC, allowing more rosette leaves to be produced. Even in the SLC when flowering is at the same time as Cvi, Bur has more rosette leaves, with 2× to 4× more rosette leaves than Cvi in the two life cycles. In contrast, Cvi rosette leaf number appears to be fixed. Therefore, Bur has a larger vegetative source tissue contributing to the reproductive sink. Furthermore, the later flowering of Bur in the WLC would result in higher light intensity to provide increased potential for photosynthesis. The restriction in vegetative source tissue may explain the more stunted growth habit of Cvi seen here.

The impact of rising CO_2_ during future climate change is not accounted for here. However, higher CO_2_ levels resulted in earlier flowering and increased seed yield in arabidopsis (Ward and Strain 1997). It is unclear if this will counter the impact of temperature on seed yield , particularly in Bur where increased temperature has a negative effect on fertility (Huang *et al*., 2014, 2018). In the wider context, flowering time determines the thermal window for seed development and maturation (Springthorpe and Penfield, 2015), with flowering time genes also contributing to seed dormancy. Of these, *FLC* in the zygotic environment reduces dormancy, but it is repressed by the autonomous flowering pathway (Chiang *et al.*, 2009; Auge *et al.*, 2018). However, in Bur, *FLC* is a null allele (Werner *et al.*, 2005) so plays no part in dormancy.

### Seedling emergence timing (SET)

Manipulation of the thermal environment during F_1_ seed production altered future life cycles. First, it altered the timing of the F_1_ reproductive phase transition (bolting time). This is consistent with later, rather than earlier, environments in the parental life cycle being better predictors of progeny environments (Auge *et al.*, 2017). Secondly, we found that SET in the F_2_ generation responds to the environment experienced during F_1_ seed production.

In both ecotypes, warm temperatures experienced in the F_2_ generation decreased primary dormancy when germination was tested in the laboratory, consistent with earlier reports (Kendall *et al.*, 2011; Awan *et al.*, 2018; Huang *et al.*, 2018). Laboratory-based dormancy tests are blunt instruments not always suited to differentiating long-term impacts of the environment. Therefore, to further investigate the effect of intergenerational temperature, we looked at SET along the thermal gradient. When seeds were buried in spring to mimic seed dispersal following a WLC, we observed a strong influence of the intergenerational thermal memory on dormancy cycling leading to SET. If SET responded only to the seasonal soil temperatures experienced, the peak SET would have been the same regardless of the temperature experienced in the maternal environment. We show that this is not the case as maternal temperatures influenced subsequent SET.

The response of SET to soil temperature differed between Bur and Cvi. Bur seedling emergence occurred when soil temperature was rising and falling, indicating greater plasticity in this trait than in Cvi, which only emerged when soil temperature was falling, as seen previously (Footitt *et al.*, 2020). Soil temperature also had a significant impact on total seedling emergence along the thermal gradient. Total emergence in Bur decreased and in Cvi increased at higher temperatures. In both ecotypes, the soil temperature gradient (2.5 °C) between the cool and warm ends of the tunnel had a significant impact on peak SET, which was delayed at the warm end. In both ecotypes, the earliest peak in SET was at the cool end of the gradient. Increasing soil temperature delayed emergence in seeds from each growth temperature regime. This is consistent with induction (Bur), and relief (Cvi) of secondary dormancy by higher temperatures (Footitt *et al*., 2011, 2017; Huang *et al.*, 2015).

### SET in Bur

In Bur, the early peak SET at the cool end of the thermal gradient reflects greater dormancy loss under cooler conditions. As dormancy is reduced, seeds enter a shallow dormancy phase. In this state, they become receptive to environmental signals that remove the final layer of dormancy (e.g. light), and the permissive temperature range for germination increases (Finch-Savage and Footitt, 2017). Total seedling emergence was greater in the middle of the thermal gradient. The delay in peak SET at the warm end of the gradient results from secondary dormancy induction by the elevated temperature reducing the proportion of the population receptive to dormancy-breaking signals. Here, in the absence of environmental signals that remove the final layer of dormancy, secondary dormancy induction by high temperature counteracts the ability of this ecotype to germinate. The ability of Bur to germinate at the warmer temperatures may have evolved due to lack of high temperature selection pressure in the low temperature environment to which this ecotype is adapted. As seedlings emerge along the thermal gradient, the order in which seeds from the different temperature regimes reach peak SET changes until at the warm end the order of peak SET is the same as seen for Cvi at all positions along the gradient. It therefore appears that increasing soil temperature forces Bur to act more like the obligate winter annual Cvi.

### SET in Cvi

In the deeply dormant Cvi ecotype, emergence was lower than in Bur. However, a clear effect of the maternal temperature regimes emerged. At each position along the thermal gradient, the earliest and latest peak SET was in seeds with C@W and W@C thermal histories, respectively, indicating that warm temperatures in the F_2_ generation reduced dormancy, and advanced emergence and cool temperatures had the opposite effect. Seeds with W@W and C@C thermal histories had intermediate SET peaks. This indicates the impact of thermal history on seed behaviour in the field.

Overall, as temperature increased along the gradient, Cvi seeds experience temperatures that remove dormancy as seen in the field and laboratory (Footitt *et al*., 2011, 2017; Huang *et al.*, 2015). However, at these temperatures, sensitivity to spatial signals that remove the final layer of dormancy is not high enough for them to be effective; a consequence of the high temperature thermo-dormancy seen in Cvi. Only when soil temperature declines does an increasing proportion of the population become sensitive to spatial signals and seedling emergence commences. The seedling emergence phase ends as secondary dormancy is induced by decreasing soil temperature, as seen previously (Footitt *et al*., 2011, 2017).

The regulation of SET appears to be by proteins encoded by the genes *DELAY OF GERMINATION 1* (*DOG1*) and *ABA-HYPERSENSITIVE GERMINATION 1* (*AHG1*). DOG1 binds to AHG1, repressing its role in downregulating ABA signalling, resulting in loss of seed dormancy (Née *et al.*, 2017). In a screen for SET quantitative trait loci (QTLs) in a Cvi × Bur recombinant inbred line mapping population, the SET QTL with the highest LOD score (17.03) was on chromosome 5 and contained *AHG1* (Footitt *et al.*, 2020).

### Conclusions

We reveal a strong adaptive response to global warming in the winter annual ecotype Cvi, compared with a weaker response in the summer annual ecotype Bur. In Cvi, flowering time advanced at twice the rate of that of Bur in a WLC. Cvi showed significant intergenerational responses in flowering time when the F_1_ seed production was under the warmer conditions indicative of 2080. This is consistent with the operation of a thermal memory. Seedling emergence timing responded positively to increased temperature in Cvi, but negatively in Bur. This indicates that Cvi emergence would continue to occur past the peak in summer temperature so avoiding potential drought conditions. In contrast, in Bur, emergence would continue in potentially hostile environments for seedling establishment. Overall, we show that the obligate winter annual Cvi responds more positively to global warming than the obligate summer annual Bur. This confirms the prediction by Marcer *et al.*, (2018) that early flowering and deep dormancy will be selected for by a warming climate.

## SUPPLEMENTARY DATA

Supplementary data are available online at https://academic.oup.com/aob and consist of the following. Figure S1: seedling emergence of buried F_2_ seeds of Cvi and Bur seeds produced in a winter life cycle in a global warming scenario. Table S1: mean air temperature from transplanting to bolting in the secomd generation at the cool and warm ends of the thermal gradient. Table S2: mean air temperature during seed maturation in the first and second generations for seed produced at the cool and warm ends of the thermal gradient. Table S3: ecotype differences in days to peak SET (T50) in response to soil temperature along the thermal gradient. Table S4: days following burial of Bur and Cvi seeds required to reach 50 % seedling emergence along the thermal gradient. Table S5: impact of soil temperature on the days to peak SET (T50) for each intergenerational temperature regime. Data S1: statistical output of analysis of variance.

mcaa141_suppl_Supplementary_Material
