# Supplementary material for: Changes in phenological events in response to a global warming scenario reveal greater adaptability of winter annual compared with summer annual arabidopsis ecotypes
Source: Ann Bot. 2020 Jul 29;127(1):111–22. doi: 10.1093/aob/mcaa141 (PMC7750725; doi:10.1093/aob/mcaa141)
Supplement: mcaa141_suppl_Supplementary_Material [file mcaa141_suppl_supplementary_material.docx]

**SUPPLEMENTAL DATA**

**Figure S1.** Seedling emergence of buried F2 seeds of Cvi and Bur seeds produced in a winter life cycle under global warming scenarios. Final percentage seedling emergence in 2013 of (A) Cvi and (C) Bur seeds produced under different intergenerational temperature regimes. The total emergence for 2013 and 2014 is shown for (B) Cvi and (D) Bur. Analysis for 2013 only was by one way ANOVA with Bonferroni correction followed multiple comparisons for significance using Tukey’s range test. The level of significance for Cvi was (*F* _11, 24_ = 5.48, *p* < 0.00025) and for Bur (*F* _11, 24_ = 6.03, *p* = 0.00012). For each ecotype, analysis covers all intergenerational- and soil temperature combinations. Combinations identified by a different letter are significantly different. For each intergenerational temperature n = 3 at each position on the thermal gradient.


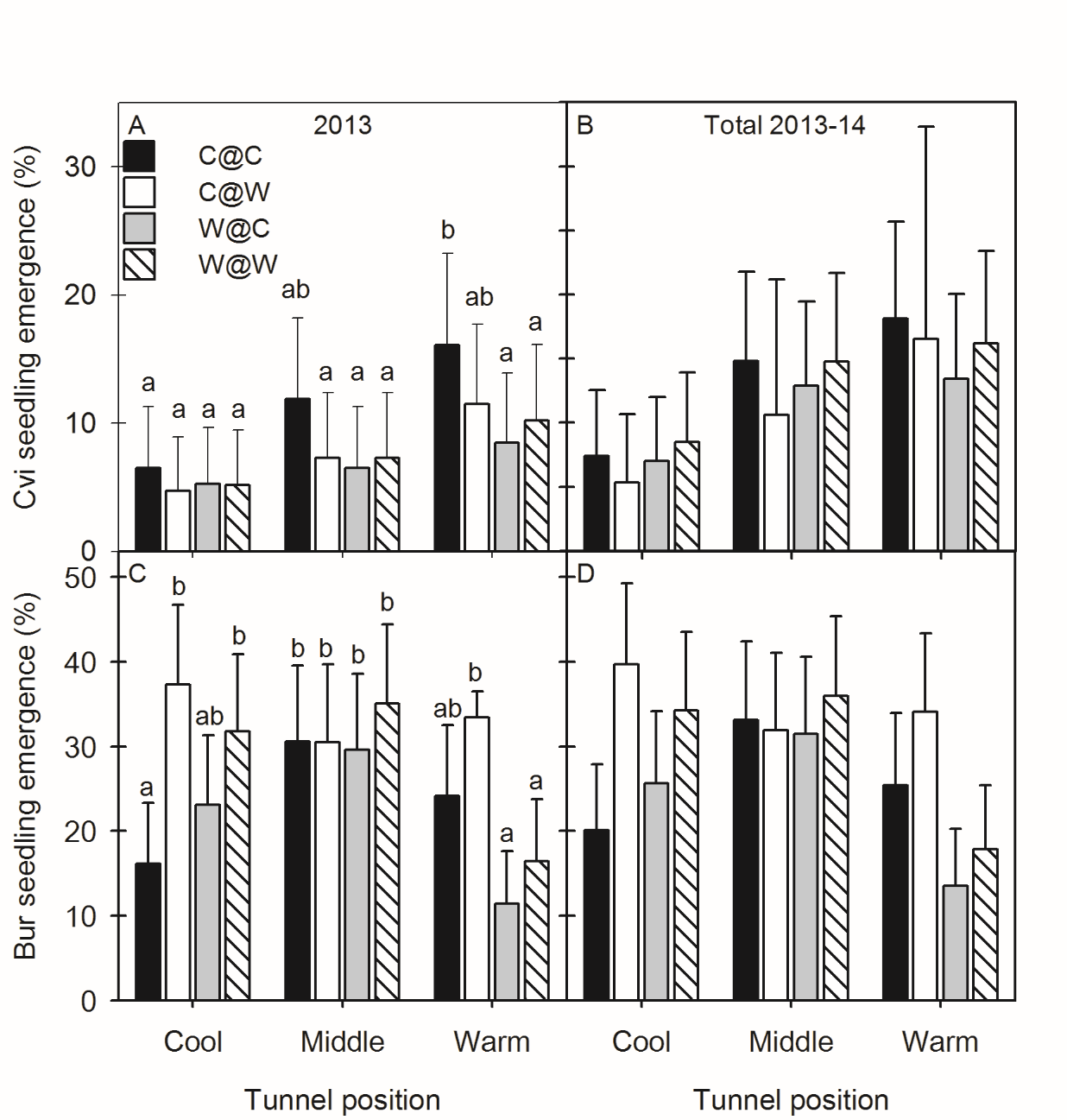


**Table S1.** Mean air temperature from transplanting to bolting in the 2^nd^ generation at the cool and warm ends of the thermal gradient. For each life cycle, the mean temperatures followed by different letters (Cvi) or *, ** (Bur) are significantly different. Analysis was by one way ANOVA with Bonferroni correction followed by multiple comparisons for significance using Tukey’s range test. In the winter life cycle the level of significance for Cvi was (*F* _3, 86_ = 23687, *p* < 0.0001) and for Bur (*F* _3, 92_ = 34565, *p* < 0.0001) and in the summer life cycle the level of significance for Cvi was (*F* _3, 92_ = 4584, *p* < 0.0001) and for Bur (*F* _3, 92_ = 27565, *p* < 0.0001). Data represent the mean ± standard error.

| 1st Generation temperature | 2^nd^ Generation life cycle | 2^nd^ Generation  mean air temperature to bolting (°C) | |
| --- | --- | --- | --- |
|  | Winter life cycle | Cool | Warm |
| Cool | Cvi | 4.89 ± 0.01 a | 8.50 ± 0.02 b |
| Warm | Cvi | 4.91 ± 0.01 a | 8.49 ± 0.01 b |
| Cool | Bur | 4.66 ± 0.01 * | 7.89 ± 0.01 ** |
| Warm | Bur | 4.64 ± 0.01 * | 7.90 ± 0.01 ** |
|  | Summer life cycle | Cool | Warm |
| Cool | Cvi | 12.67 ± 0.05 b | 16.57 ± 0.03 c |
| Warm | Cvi | 12.53 ± 0.02 a | 16.63 ± 0.02 c |
| Cool | Bur | 12.68 ± 0.01 * | 16.72 ± 0.01 ** |
| Warm | Bur | 12.66 ± 0.02 * | 16.74 ± 0.01 ** |

**Table S2.** Mean air temperature during seed maturation in the 1^st^ and 2^nd^ generations for seed produced at the cool and warm ends of the thermal gradient. In each generation, maturation temperature was calculated as the mean air temperature over 30 days prior to harvest except for Cvi in the 2^nd^ generation at the warm end of the gradient where time from last plant bolting to harvest in the summer life cycle was 26 (C@W) and 27 days (W@W). For each life cycle, the mean temperatures followed by different letters (Cvi) or *, ** (Bur) are significantly different. Analysis was by one way ANOVA with Bonferroni correction followed by multiple comparisons for significance using Tukey’s range test. In the winter life cycle the level of significance for Cvi was (*F* _3, 116_ =10, *p* < 0.0001) and for Bur (*F* _3, 116_ = 18, *p* < 0.0001) and in the summer life cycle the level of significance for Cvi was (*F* _3, 111_ = 0.85, *p* = 0.46) and for Bur (*F* _3, 116_ = 11, *p* < 0.0001).Data represent the mean ± standard error. Seed maturation temperatures for the 1^st^ generation are as in Huang *et al*., (2018).

| 1^st^ Generation  Seed maturation temperature (°C) | | 2^nd^ Generation life cycle | 2^nd^ Generation  Seed maturation temperature (°C) | |
| --- | --- | --- | --- | --- |
| Mean over 30 days prior to harvest | |  | Mean over 30 days prior to harvest | |
| Winter life cycle | | Winter life cycle | Cool | Warm |
| Cool | 12.96 ± 0.43 | Cvi | 12.33 ± 0.46 a | 14.85 ± 0.45 b |
| Warm | 17.02 ± 0.49 | Cvi | 12.33 ± 0.46 a | 14.85 ± 0.45 b |
| Cool | 13.39 ± 0.28 | Bur | 12.46 ± 0.43 * | 15.47 ± 0.38 ** |
| Warm | 17.22 ± 0.46 | Bur | 12.46 ± 0.43 * | 15.52 ± 0.38 ** |
|  |  | Summer life cycle |  |  |
| Cool | 12.96 ± 0.43 | Cvi | 19.81 ± 0.54 a | 20.67 ± 0.48 a |
| Warm | 17.02 ± 0.49 | Cvi | 19.81 ± 0.54 a | 20.59 ± 0.47 a |
| Cool | 13.39 ± 0.28 | Bur | 18.53 ± 0.58 * | 21.76 ± 0.53 ** |
| Warm | 17.22 ± 0.46 | Bur | 18.53 ± 0.58 * | 21.76 ± 0.53 ** |

**Table S3.** Days following burial of Bur and Cvi seeds required to reach 50% seedling emergence along the thermal gradient*.* Seedling emergence of seeds produced under different intergenerational temperature regimes. Burial of seeds produced in a winter annual life cycle in the 2^nd^ generation was at three positions in a thermal gradient tunnel. Times indicate days from the last burial date for Bur and Cvi (see methods) to the 50% seedling emergence determined by performing Probit transformation of the data and linear regression analysis of each replicate. Analysis was by one way ANOVA with Bonferroni correction followed by multiple comparisons for significance using Tukey’s multiple range test. The ANOVA generated *F* statistics are as follows; for tunnel position Bur (*F* _2, 33_ = 8.55, *p* = 0.0010), Cvi (*F* _2, 33_ = 6.97, *p* = 0.0029); all tunnel positions Bur (*F* _3, 32_ = 2.5, *p* = 0.075), Cvi (*F* _3, 32_ =8.2, *p* =0.0003); Cool Bur (*F* _3, 8_ = 3.86, *p* = 0.055), Cvi (*F* _3, 8_ =3.44, *p* =0.071); Middle Bur (*F* _3, 8_ =5.94, *p* < 0.0197), Cvi (*F* _3, 8_ =5.9, *p* < 0.0197); Warm Bur (*F* _3, 8_ = 3.26, *p* < 0.08), Cvi (*F* _3, 8_ = 13.6, *p* < 0.0016). In each column values followed by a different letters are significantly different; for tunnel position significance is denoted by the symbols * and #.

| Inter- generational temperature regime | Days to peak SET (T50) in Bur at each tunnel position | | | |
| --- | --- | --- | --- | --- |
|  | All positions | Cool | Middle | Warm |
| C@C | 65.5 ± 1.8 a | 62.5 ± 3.1 b | 63.9 ± 3.0 ab | 70.0 ± 2.2 a |
| C@W | 72.2 ± 5.6 a | 38.8 ± 4.6 a | 66.7 ± 7.5 ab | 67.6 ± 3.9 a |
| W@C | 57.7 ± 5.5 a | 56.7 ± 8.5 ab | 72.2 ± 5.4 b | 87.7 ± 6.1 a |
| W@W | 56.9 ± 4.2 a | 55.8 ± 2.3 ab | 44.9 ± 0.1 a | 69.9 ± 7.0 a |
| Tunnel position |  | 53.5 ± 3.5 * | 61.9 ± 3.7 *# | 73.8 ± 3.3 # |
| Inter- generational temperature regime | **Days to peak SET (T50) in Cvi at each tunnel position** | | | |
|  | All positions | Cool | Middle | Warm |
| C@C | 75.1 ± 2.8 a | 68.9 ± 2.8 a | 72.8 ± 5.0 ab | 83.6 ± 0.3 a |
| C@W | 70.9 ± 1.8 a | 66.3 ± 1.7 a | 68.4 ± 0.7 a | 77.9 ± 0.5 a |
| W@C | 85.5 ± 1.5 b | 80.6 ± 2.2 a | 86.0 ± 0.8 b | 90.0 ± 1.0 b |
| W@W | 78.5 ± 1.8 ab | 74.9 ± 4.0 a | 78.5 ± 0.5 ab | 82.2 ± 1.9 a |
| Tunnel position |  | 72.7 ± 2.2 * | 76.4 ± 2.48 *# | 83.4 ± 1.4 # |

**Table S4. Impact of soil temperature on the days to peak SET (T50) for each intergenerational temperature regime.** Data in table S2 was analysed by one way ANOVA with Bonferroni correction followed by multiple comparisons for significance using Tukey’s multiple range test. The ANOVA generated values of the *F* statistic are given in the table. In each row values followed by a different letters are significantly different.

| Inter- generational temperature regime | Significant differences in peak SET (T50) in Bur in response to soil temperature along the thermal gradient | | | | | | |
| --- | --- | --- | --- | --- | --- | --- | --- |
|  | ANOVA generated *F* statistic | | Cool | | Middle | Warm |  |
| C@C | (*F* _2, 6_ = 2.05, *p* < 0.209) | | a | | a | a |  |
| C@W | (*F* _2, 6_ = 8.65, *p* < 0.016) | | a | | b | b |  |
| W@C | (*F* _2, 6_ = 5.21, *p* < 0.048) | | a | | ab | b |  |
| W@W | (*F* _2, 6_ = 8.62, *p* < 0.017) | | ab | | a | b |  |
| Inter- generational temperature regime | **Significant differences in** **peak SET (T50) in Cvi in response to soil temperature along the thermal gradient** | | | | | | |
|  | ANOVA generated *F* statistic | Cool | | Middle | | Warm | |
| C@C | (*F* _2, 6_ = 3.48, *p* < 0.098) | a | | a | | a | |
| C@W | (*F* _2, 6_ = 7.22, *p* < 0.025) | ab | | b | | a | |
| W@C | (*F* _2, 6_ = 7.11, *p* < 0.026) | a | | ab | | b | |
| W@W | (*F* _2, 6_ = 1.33, *p* < 0.331) | a | | a | | a |  |

**Table S5. Ecotype differences in days to peak SET (T50) in response to soil temperature along the thermal gradient.** Data for the T50 SET for each ecotype along the thermal gradient was analysed to determine differences in response to soil temperature along the thermal gradient. For ‘all positions’ data for each ecotype was compared. Then Mean peak SET was compared to identify significant differences between ecotypes and tunnel position. Analysis was by one way ANOVA with Bonferroni correction followed by multiple comparisons for significance using Tukey’s multiple range test. For each ecotype (all positions) the ANOVA generated the *F* statistic was (*F* _1, 70_ = 26.98, *p* < 0.0001). For differences between ecotypes and positions on the gradient the ANOVA generated the *F* statistic was (*F* _5, 66_ = 14.11, *p* < 0.0001). Means followed by a different letter or symbol are significantly different.

|  | **Mean time to peak SET (T50) at each position along the thermal gradient in the Bur and Cvi ecotypes.** | | | |
| --- | --- | --- | --- | --- |
|  | **Tunnel Positon** | | | |
| **Ecotype** | **All positions** | **Cool** | **Middle** | **Warm** |
| **Bur** | 63.1 ± 2.4 * | 53.4 ± 3.5 a | 61.9 ± 3.7 ac | 73.8 ± 3.3 bc |
| **Cvi** | 77.5 ± 1.3** | 72.7 ± 2.2 bcd | 76.4 ± 2.4bd | 83.4 ± 1.4 bd |
